# Kawasaki Disease Complicated by *Salmonella oranienburg* Coinfection

**DOI:** 10.1155/2021/5584514

**Published:** 2021-04-02

**Authors:** Zachary Barbara, Savannah P. Ellenwood, Emily D. Loe, Joon Choi, Kathleen Ryan, Nancy Joseph

**Affiliations:** ^1^University of Florida Shands Children's Hospital, Department of Pediatrics, Gainesville, FL, USA; ^2^University of Florida, School of Medicine, Gainesville, FL, USA; ^3^University of Florida Shands Children's Hospital, Department of Pediatric Hospital Medicine, Gainesville, FL, USA

## Abstract

Kawasaki disease is a medium vessel vasculitis with a multisystem presentation affecting 9–20 per 100,000 children under 5 years of age in the United States. *Salmonella* coinfection has not been previously described. We present a 12-month-old vaccinated male with Kawasaki disease in the setting of *Salmonella* bacteremia. Initial intervention for the Kawasaki disease with IVIG was ineffective, prompting adjunctive therapy with anakinra, with eventual full recovery. Concurrent Kawasaki disease and bacteremia may confound diagnosis and necessitate nontraditional treatment approaches.

## 1. Introduction

The classic diagnostic criteria of Kawasaki disease (KD) includes at least 5 days of fever and the presence of at least 4 clinical criteria, including bilateral bulbar conjunctival injection, diffuse maculopapular rash, cervical lymphadenopathy, oral mucous membrane changes, and peripheral extremity changes [1, 2]. If left untreated, it can be a self-limiting disease that resolves in 12 days, on average. However, the cardiovascular complications, including giant coronary artery aneurysms, can cause significant morbidity and mortality. Concomitant infections in patients with KD rarely occur, but have been reported with adenovirus, rotavirus, respiratory viruses, and *Streptococcus pneumoniae*, but not *Salmonella* [2, 3]. Coinfections occur more with incomplete Kawasaki or IVIG-resistant Kawasaki.

*Salmonella* species are motile Gram-negative rods with multiple serotypes that can cause a fever syndrome known as typhoid fever, as well as gastroenteritis, bacteremia, and osteomyelitis. Typhoidal *Salmonella* presents with high “stepwise” fever and development of bacteremia in the first week. The classic “rose spots” and abdominal pain present in the second week of illness [4, 5]. The third week comprises hepatosplenomegaly, intestinal bleeding, and possible perforation due to Peyer's patch hyperplasia [4, 5].

Less than 1% of bacteremia caused by *Salmonella* is due to nontyphoidal *Salmonella* [4, 6]. Previously healthy children less than 5 years of age bear the largest disease burden [4, 6]. Fulminant disease can result in septic shock and death [4, 6]. Conversely, in the absence of critical complications, the majority of patients will recover over weeks to months [4–6].

We present a case of Kawasaki disease with IVIG resistance in the setting of *Salmonella* bacteremia.

## 2. Case Summary

A previously healthy, vaccinated 12-month-old male who was born full term presented to our hospital with two and a half days of fever, rash, red eyes, and nonbloody diarrhea. The rash started behind the ears and then spread to his entire body. He was breast- and bottle-fed, with no history of prior hospitalizations or day care exposures. He lived at home with his biological parents and three older male siblings.

Upon presentation, the patient was febrile up to 102.6 F, with a blood pressure of 110/77 mmHg and heart rate of 174 beats/min.

Initial assessment revealed an interactive, well-appearing infant with an erythematous tongue, cracked lips, bilateral nonpurulent scleral injection with limbic sparing, hand and foot swelling, and diffuse polymorphous rash sparing the palms and soles. He was noted to have a 1.5 cm mobile, nontender anterior cervical lymph node. Head examination was normocephalic, atraumatic, and revealed a closed anterior fontanelle. Cardiovascular, respiratory, and abdominal examinations were unremarkable.

Initial laboratory data revealed an elevated CRP of 115 mg/L, elevated ESR to 55 mm/hour, and no leukocytosis. Chemistry was pertinent for slightly increased ALT to 60 IU/L but normal alkaline phosphatase and AST. Respiratory viral panel PCR was positive for rhino/enterovirus, and his SARS CoV-2 PCR was negative. His presentation was concerning for evolving Kawasaki disease versus a viral illness, so he was admitted for monitoring of fever, as five days of fever along with his clinical findings would meet the diagnostic criteria for KD.

The patient continued to be febrile throughout the next two days with his signs of illness waxing and waning. Blood culture was obtained and was negative. A GI PCR panel was sent due to persistent diarrhea, which was positive for enteropathogenic *E. coli* and unspecified *Salmonella* species. A transthoracic echocardiogram was obtained on the fifth day of fever showing no evidence of coronary artery dilation. By the seventh day, his clinical exam was unchanged, and the echocardiogram was repeated due to persistent fever. The proximal right and left main coronary arteries were noted to be at the upper limit of normal with a Z-score of 1.9 and concern for “beads-on-a-string” appearance of the left main coronary artery. The left anterior descending artery (LAD) was moderately dilated with a Z-score of 3.27, meeting criteria for a small aneurysm. Treatment with 2 g/kg IVIG and 80 mg/kg/day of aspirin was started.

**Figure 1:**
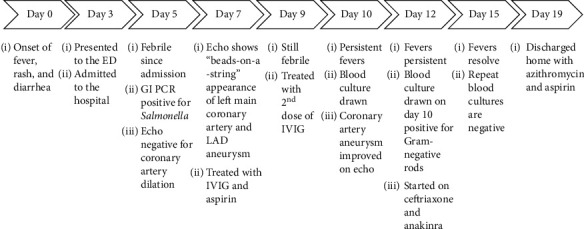
Representation of the patient's clinical course showing the main diagnostic and treatment decision points.

48 hours after IVIG, there was recrudescence of the fever, suggesting resistance to IVIG treatment. This led to administration of a second dose of IVIG on the ninth day of illness. After this second round of IVIG, a third echo showed that his coronary artery aneurysms had improved. At this time, his clinical symptoms also began improving. However, he continued to have daily fevers ranging from 38–39.5° C. On day 12, the blood culture that was drawn on the tenth day of illness was noted to be positive for Gram-negative rods, and he was started on IV ceftriaxone. An ultrasound of the abdomen was obtained and found to be unremarkable. At this juncture, pediatric rheumatology was consulted for IVIG-resistant KD and recommended treatment with 8 mg/kg/day of anakinra (an IL-1 receptor antagonist). Eventual state laboratory speciation revealed pansensitive *Salmonella oranienburg*. Three days after initiation of antibiotics, the fevers resolved, and repeat blood cultures were negative. He was treated with ceftriaxone for seven days and discharged with oral azithromycin for an additional five days. Upon follow-up, the patient was doing well without further complications or recurrence of symptoms. Follow-up echocardiography showed resolution of the coronary artery aneurysms. This timeline is reviewed in Figure 1.

## 3. Discussion

The etiology of Kawasaki disease (KD) is largely unknown, but numerous studies have indicated that it is not infectious in nature, despite KD having many properties supportive of infectious causation [2, 7, 8]. Additionally, Yanagawa et al. described a “wave” of KD cases in Japan during the winter of 1985–1986 that was typical of spread via respiratory viral illness [7, 9]. Epidemiologically, KD has been reported to have a higher incidence during the winter to spring months worldwide, again coinciding with respiratory viral seasons [7, 8, 10].

The 2017 AHA statement on Kawasaki Disease reports that there is no known pathogenesis of this vasculitis [11]. Recently, however, there have been reports suggestive of a relationship between KD and the gut microbiome, but with few reports of potential organisms. One proposed mechanism is a relationship between gastrointestinal flora that utilize lipopolysaccharide (LPS) binding protein, a protein that is utilized by *Salmonella* serotypes [12]_._ When looking at serum LPS binding protein, LPS and superantigens have been found in higher quantities in individuals with KD than even those with bacterial sepsis [12–15]. In addition, genomic analysis of the gut microbiome of affected patients has also found disruptions in normal flora during acute-phase KD [13]. There is speculation that this gastrointestinal disruption could stimulate the pathological immune disruption seen in KD [14].

Coinfections have been reported with KD, mostly in relation to IVIG-resistant KD [2, 8, 16]. Some common viruses including adenovirus, rhino/enterovirus, respiratory syncytial virus, influenza, and parainfluenza have been reported [2, 8, 16, 17]. In this case, the patient was noted to be rhino/enterovirus-positive on the respiratory virus panel with no symptoms of rhinorrhea or congestion. This likely indicates shedding from a previous infection and did not contribute to his IVIG resistance. Bacteria such as *Staphylococcus aureus*, *Streptococcus pyogenes*, *Mycoplasma pneumoniae*, and *Chlamydia pneumoniae* have been reported, as well [2]. To our knowledge, this is the first time *Salmonella* has been reported in a patient with KD. We postulate that the presence of *Salmonella* coinfection was the cause of the persistent fever and assumption of IVIG resistance.

*Salmonella* species are passed to humans by contaminated food or surfaces or pets. The bacteria survive in the gastrointestinal tract by producing cytotoxins [18]. Most infections cause gastroenteritis with or without fever, nonbloody diarrhea, and stomach cramps; however, in certain patient populations, including those with weakened immune systems, sickle cell disease, and children less than 5 years of age, infection can become disseminated [19]. Severe forms of *Salmonella* infection include bacteremia, meningitis, osteomyelitis, and enteric fever. Usually, gastroenteritis does not require treatment, as antibiotic intervention can prolong the carrier state and increase antibiotic resistance. However, systemic infections should be treated with antibiotics, as severe infections can lead to significant morbidity and mortality [18].

*Salmonella oranienburg* is a serotype of *Salmonella* known to cause widespread outbreaks of gastroenteritis linked to chocolate consumption, biofilms on eggs, and pet turtles [19–21]. It causes about ten percent of all clinical *Salmonella* infections [22]. In the patient's county of origin, the rate of *Salmonella oranienburg* infection was 0.01 to 0.15 per 100,000 people. He was known to have a pet turtle, which was likely the origin of his infection. Only 7.7 percent of cases of *Salmonella oranienburg* cause blood, CSF, or joint infections. The rate of infection was consistently highest among children aged 0 to 4 years from 1968 to 2011 [19].

Regardless of the concomitant agent, it has been thought that coinfections present a dilemma as it pertains to diagnosing and treating KD [8, 23–25]. Evidence has suggested that delaying therapy for KD is associated with increased risk of treatment failure with IVIG and development of coronary artery aneurysms [8, 26]. In light of this risk, multiple studies have recommended a low threshold for initiating IVIG therapy in children with documented respiratory viral illness and incomplete or evolving clinical KD criteria [2, 8, 16, 24]. Despite no delay in treatment when the patient met criteria, he remained persistently febrile which was deemed as IVIG resistance. In hindsight, *Salmonella* bacteremia may have been the confounding variable throughout his clinical course.

Categorization of a patient as an “IVIG nonresponder” or diagnosing “refractory Kawasaki disease” is generally thought to coincide with persistent or recrudescent fever >37.5^◦^C in 24–48 hours following IVIG treatment [23]. This definition varies slightly in the literature based on the type of risk scoring system being utilized. However, there is one clear consensus that IVIG nonresponders are at increased risk of coronary artery aneurysms [23, 27]. In addition, a collaborative retrospective study in Italy found that patients with “abdominal manifestations” of Kawasaki disease were more likely to be resistant to IVIG and develop coronary artery aneurysms [28].

Downie et al. reported that patients with documented infection were more likely to have partial or complete nonresponse to IVIG, necessitating second-line therapy or additional doses of IVIG [23]. Additionally, complete nonresponders were at higher risk for developing coronary artery aneurysms [23]. Our patient would have been classified as a partial responder, as during initial IVIG treatment, his temperature decreased to <37.5° C, but then subsequently recurred. His concomitant *Salmonella* gastroenteritis in the presence of Kawasaki disease vasculitis could have allowed for translocation of bacteria into the bloodstream.

This case provided a complex clinical picture. At the time of initial presentation, this patient met criteria for incomplete KD. But in the setting of positive RVP and GI PCR with organisms that have been shown to have some correlation with KD, the patient was admitted for clinical monitoring of evolving KD. Once the patient met clinical criteria with positive echo findings, treatment with IVIG and aspirin was initiated. Regarding his *Salmonella* infection, the patient did not meet treatment criteria for *Salmonella* enteritis [6]. His initial blood culture was negative, and when repeat blood cultures showed *Salmonella* bacteremia later in his course, treatment was necessitated. We theorized that the likelihood of translocation was increased due to the enhanced permeability throughout the GI tract due to KD and enteritis.

## 4. Conclusion

We present, to our knowledge, the first case of Kawasaki disease complicated by concurrent *Salmonella* bacteremia. This case presented multiple diagnostic and treatment dilemmas that provided an interesting course on how to approach Kawasaki disease in the setting of multiple cofounders. We hope that the experience gained from this case can serve to inform treatment of concomitant infections in the setting of Kawasaki disease. Furthermore, it highlights the evidence that supports keeping Kawasaki disease on the differential in the setting of concomitant disease processes.

## Data Availability

The personal health data used to support the findings of this study are restricted by the University of Florida Office of Compliance and Ethics in order to protect patient privacy. Data are available from University of Florida Department of Compliance and Ethics at email: UF-Compliance@ufl.edu or phone: (352) 294 8720 for researchers who meet the criteria for access to confidential data.
